# Evaluation of Antioxidative and Cytotoxic Activities of *Streptomyces pluripotens* MUSC 137 Isolated from Mangrove Soil in Malaysia

**DOI:** 10.3389/fmicb.2015.01398

**Published:** 2015-12-16

**Authors:** Hooi-Leng Ser, Nurul-Syakima Ab Mutalib, Wai-Fong Yin, Kok-Gan Chan, Bey-Hing Goh, Learn-Han Lee

**Affiliations:** ^1^Biomedical Research Laboratory, Jeffrey Cheah School of Medicine and Health Sciences, Monash University MalaysiaBandar Sunway, Malaysia; ^2^UKM Medical Molecular Biology Institute–UKM Medical Centre, Universiti Kebangsaan MalaysiaKuala Lumpur, Malaysia; ^3^Division of Genetics and Molecular Biology, Institute of Biological Sciences, Faculty of Science, University of MalayaKuala Lumpur, Malaysia

**Keywords:** *Streptomyces pluripotens*, cytotoxic, antioxidative, mangrove, actinobacteria

## Abstract

*Streptomyces pluripotens* MUSC 137 was isolated from mangrove soil obtained from Tanjung Lumpur, Pahang, Malaysia. We investigated the phylogenetic, genomic, biochemical, and phenotypic characteristics of this strain. Uniquely adapted microorganisms from mangrove habitats have previously yielded compounds of biopharmaceutical interest. In order to examine the bioactivities possessed by the strain, fermentation extract was prepared through solvent extraction method prior to bioactivities screenings. Antioxidant activity was examined via DPPH assay while the cytotoxic effect was assessed by means of examining the activity of the extract against selected human cancer cell lines, namely colon cancer cells (HCT-116, Caco-2, SW480, and HT-29), breast cancer cell (MCF-7), lung cancer cell (A549), prostate cancer cell (DU145), and cervical cancer cell (Ca Ski). The results revealed MUSC 137 possesses significant antioxidant activity and demonstrates cytotoxic effect against several cancer cell lines tested. The results indicated MCF-7 cells were most susceptible to the extract with the lowest IC_50_ (61.33 ± 17.10 μg/mL), followed by HCT-116 and A549. Additionally, selective index (SI) showed that MUSC 137 extract was less toxic against normal cell lines when compared to MCF-7 and HCT-116 cells. The extract was further subjected to chemical analysis using GC–MS and revealed the presence of deferoxamine and pyrrolizidines related compounds which may account for the antioxidant and cytotoxic properties observed.

## Introduction

Mangrove is a special woody plant area of intertidal coasts in tropical and subtropical coastal regions. Mangrove ecosystems are habitats of various flora and fauna of marine, freshwater, and terrestrial species (Jennerjahn and Ittekkot, 2002). The constant changes in salinity and tidal gradient in the mangrove ecosystem have become driving forces for metabolic pathway adaptations that could direct to the production of useful metabolites (Hong et al., 2009; Lee et al., 2014a). Therefore, the growing interest in the utilization of mangrove microorganism resources have indirectly led to the discovery of *Streptomyces* from the different mangrove environments globally, such as the isolation of *Streptomyces sanyensis* (Sui et al., 2011), *S. shenzhenensis* (Hu et al., 2011), *S. qinglanensis* (Hu et al., 2012), *S. pluripotens* (Lee et al., 2014b), *S. gilvigriseus* (Ser et al., 2015a), and *S. mangrovisoli* (Ser et al., 2015b).

The genus *Streptomyces* was proposed by Waksman and Henrici (1943) and at the time of writing (July 2015), the genus *Streptomyces* is comprised of ca. 600 species with validly published names^1^ Over the years, through traditional cultivation methods, many of the bioactive compounds have been isolated from *Streptomyces* species, including antibacterial, antifungal, antioxidant, and antitumor compounds (Hong et al., 2009; Qin et al., 2011; Kawahara et al., 2012; Lee et al., 2014a,b; Kumar et al., 2014; Tan et al., 2015). The importance of these organisms is clearly seen from the fact that approximately 70% of the antibiotics in use were derived from actinobacteria (Subramani and Aalbersberg, 2012), of which 75% were derived from genus *Streptomyces* (Berdy, 2005; Rehm et al., 2009; Crnovcic et al., 2013).

In our screening program to explore the potential biological activity possessed by the strain MUSC 137, the extract was prepared and the free radical scavenging assay was used to evaluate the antioxidant activity. Different type of human cancer cell lines derived from colon, breast, cervical, prostate, and lung were selected for the screening of cytotoxic activity. These results indicated that MUSC 137 extract exhibited a significant antioxidant and cytotoxic properties. The extract was then subjected to chemical analysis with the use of GC–MS, which eventually led to the identification of chemical constituents present in the extract of MUSC 137. Taken all together, the well characterized strain, *S. pluripotens* MUSC 137 isolated from mangrove soil in Tanjung Lumpur could be a potential good source of antioxidative and chemopreventive agents.

## Materials and Methods

### Sample Collection and Isolation of Strain *Streptomyces pluripotens* MUSC 137

Strain MUSC 137 was isolated from a soil sample collected in December 2012 from site MUSC-TLS4 (3° 48′ 21.3″ N 103° 20′ 3.3″ E) located in the Tanjung Lumpur mangrove forest in the state of Pahang, Peninsular Malaysia. Topsoil samples of the upper 20-cm layer (after removing the top 2–3 cm) were collected and sampled into sterile plastic bags using an aseptic metal trowel, and stored at –20°C. Air-dried soil samples were ground with a mortar and pestle. Selective pretreatment of soil samples was performed using wet heat in sterilized water (15 min at 50°C; Takahashi et al., 1996). Five grams of the pretreated air-dried soil was mixed with 45 ml sterilized water and mill ground, spread onto the isolation medium ISP 2 (Shirling and Gottlieb, 1966) supplemented with cycloheximide (25 μg/ml) and nystatin (10 μg/ml), and incubated at 28°C for 14 days. Pure cultures of strain MUSC 137 were maintained on slants of ISP 2 agar at 28°C and as glycerol suspensions (20%, v/v) at –20°C.

### Genomic and Phylogenetic Characterization

**FIGURE 1:**
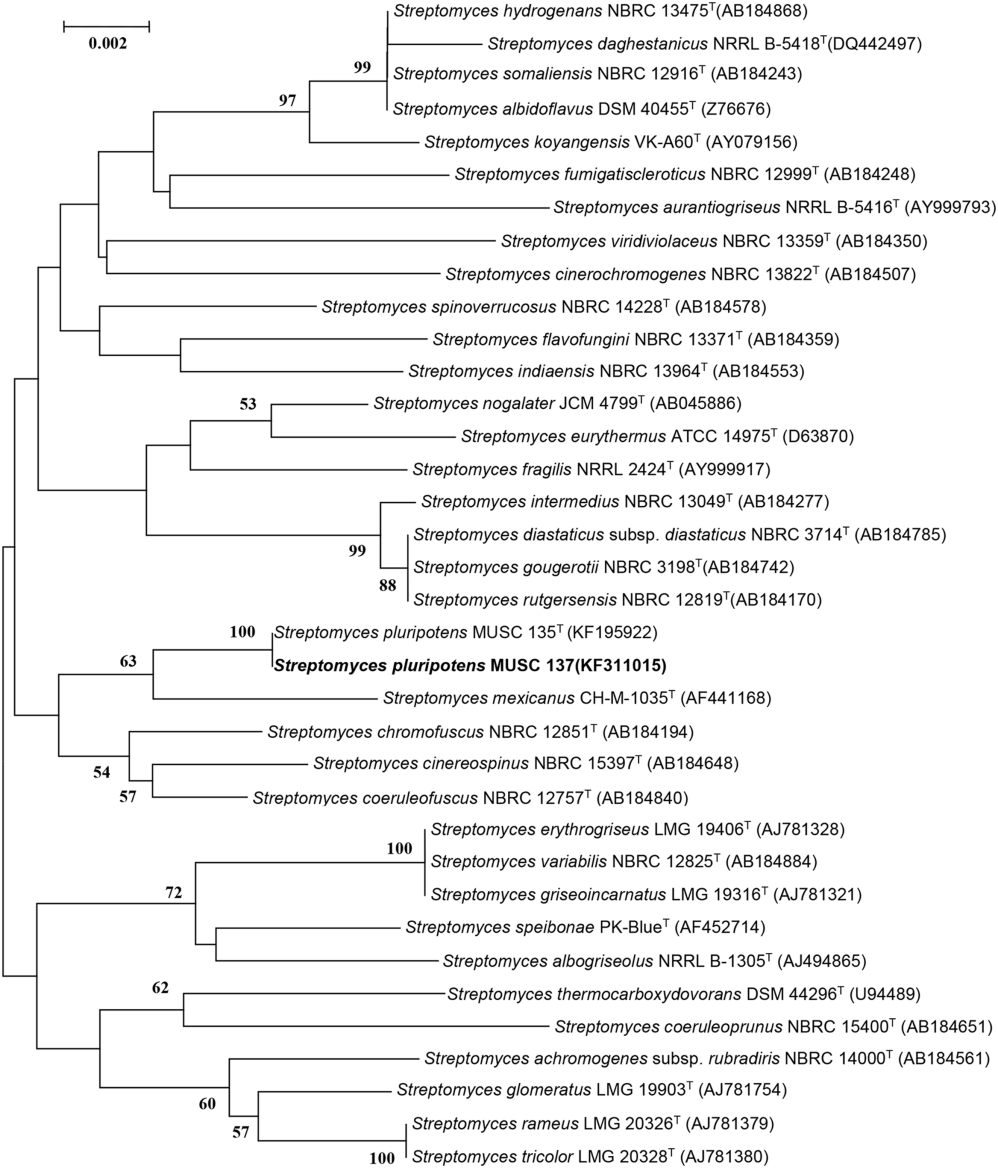
**Maximum-likelihood phylogenetic tree based on almost complete 16S rRNA sequences (1491 nucleotides) showing the relationship between strain MUSC 137 and representatives of some other related taxa.** Numbers at nodes indicate percentages of 1000 bootstrap re-samplings, only values above 50% are shown. Bar, 0.002 substitutions per site.

The bioactive-producing strain *S. pluripotens* MUSC 137 was characterized based on phylogenetic, genomic, biochemical, and phenotypic approaches. The extraction of genomic DNA and PCR was performed as described by Hong et al. (2009). The amplification of 16S rRNA gene was performed according to Lee et al. (2014b). The 16S rRNA gene sequence of strain MUSC 137 was aligned with representative sequences of related type strains of the genus *Streptomyces* retrieved from the GenBank/EMBL/DDBJ databases using CLUSTAL-X software (Thompson et al., 1997). The alignment was verified manually and adjusted prior to the reconstruction of phylogenetic trees. Phylogenetic trees were constructed with the maximum-likelihood algorithm (Felsenstein, 1981), (**Figure 1**) using MEGA version 6.0 (Tamura et al., 2013). The EzTaxon-e server^2^ (Kim et al., 2012) was used for calculation of sequence similarity. The stability of the resultant trees topologies were evaluated by using the bootstrap resampling method of Felsenstein (1985). The BOX-PCR fingerprint analysis was used to characterize strain MUSC 137 and the closely related strains using the primer BOX-A1R (5′-CTACGGCAAGGCGACGCTGACG-3′; Lee et al., 2014c). The PCR condition for BOX-PCR was performed as described by Lee et al. (2014d). The PCR products were visualized by 2% agarose gel electrophoresis.

The genomic DNA extraction for DNA-DNA hybridization of strain MUSC 137 with *S. pluripotens* MUSC135^T^ was performed according to protocol of Cashion et al. (1977). The DNA–DNA hybridization was carried out by the Identification Service of the DSMZ, Braunschweig, Germany following the protocol of De Ley et al. (1970) under consideration of the modifications described by Huss et al. (1983).

### Phenotypic Characterization

**FIGURE 2:**
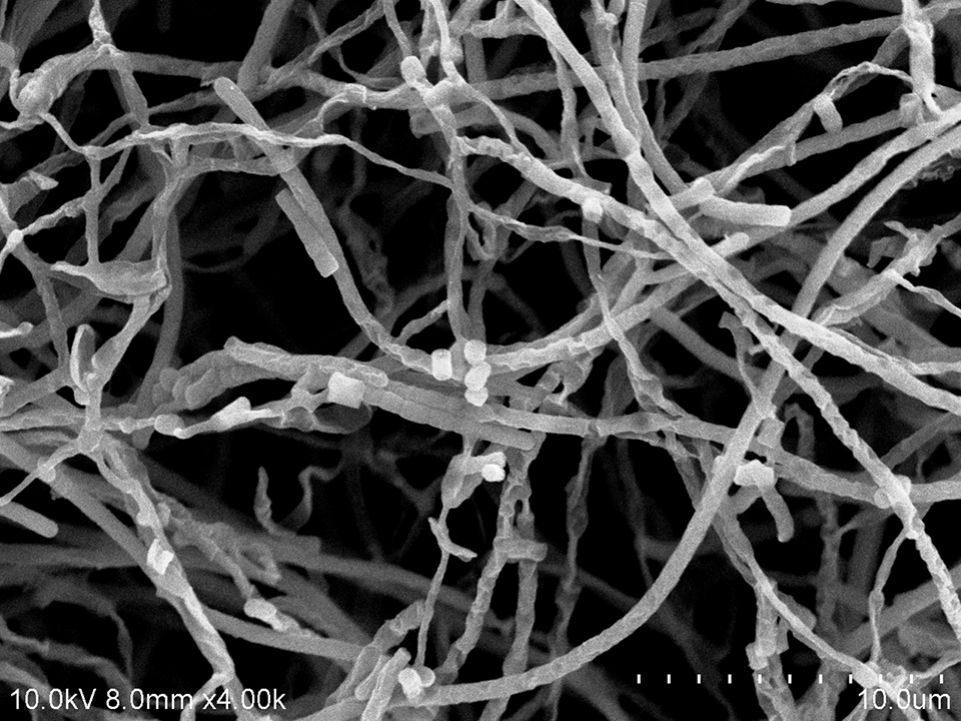
**Scanning electron microscope of *Streptomyces pluripotens* MUSC 137**.

The cultural characteristics of strain MUSC 137 were determined following growth on ISP2 and ISP7 agar (Shirling and Gottlieb, 1966), starch casein agar (SCA; Küster and Williams, 1964), *Streptomyces* agar (SA; Atlas, 1993), Actinomycetes isolation agar (AIA; Atlas, 1993), and nutrient agar (Macfaddin, 2000) for 7–14 days at 28°C. Light microscopy (80i, Nikon) and scanning electron microscopy (JEOL-JSM 6400) were used to observe the morphology of the strain after incubation on ISP2 agar at 28°C for 7–14 days (**Figure 2**). The designation of colony color was determined by using the *ISCC-NBS* color charts (Kelly, 1964). Gram staining was performed by standard Gram reaction and confirmed by using KOH lysis (Cerny, 1978). The NaCl tolerance [0–10% (w/v)] and pH range (pH 4.0–10.0) for growth was tested in TSB. The growth temperature range was tested at 4–40°C at intervals of 4°C on ISP2 agar. The responses to temperature, pH and NaCl were observed for 14 days. Hemolytic activity was assessed on blood agar medium containing 5% (w/v) peptone, 3% (w/v) yeast extract, 5% (w/v) NaCl, and 5% (v/v) horse blood (Carrillo et al., 1996). The plates were examined for hemolysis after incubation at 28°C for 7–14 days. Catalase activity and production of melanoid pigments were determined following protocols described by Lee et al. (2014e). Amylolytic, cellulase, chitinase, lipase, protease, and xylanase activities were determined on ISP2 agar as described by Meena et al. (2013). Antibiotic susceptibility tests were performed by the disk diffusion method as described by Shieh et al. (2003). Carbon-source utilization and chemical sensitivity assays were determined using Biolog GenIII MicroPlates (Biolog, USA) according to the manufacturer’s instructions. Antimicrobials used and their concentrations are as follows: ampicillin (10 μg), ampicillin sulbactam (30 μg), cefotaxime (30 μg), cefuroxime (30 μg), cephalosporin (30 μg), chloramphenicol (30 μg), ciprofloxacin (10 μg), erythromycin (15 μg), gentamicin (20 μg), nalidixic acid (30 μg), Penicillin G (10 μg), streptomycin (10 μg), tetracycline (30 μg), and vancomycin (30 μg).

### Extract Preparation of MUSC 137

Strain MUSC 137 was grown in seed medium (TSB) for 14 days prior to fermentation. The fermentation medium, FM3 was sterilized by autoclaving at 121°C for 15 min prior to experiment (Hong et al., 2009; Lee et al., 2014a). Fermentation was performed in shaking 500 mL Erlenmeyer flask containing 200 mL of FM3, for 7–10 days at 28°C. The resulting FM3 medium was recovered by centrifugation at 12000 *g* for 15 min. The supernatant was filtered and subjected to freeze dry process. Once freeze-dried, the sample was extracted thrice with methanol. The extracting solvent was removed and concentrated by rotary vacuum evaporator at 40°C. The extract of MUSC 137 was collected and suspended in dimethyl sulfoxide (DMSO) as vehicle reagent prior to assay.

### Investigation of Antioxidant Activity of MUSC 137 Extract Using 2,2-Diphenyl-1-Picrylhydrazyl (DPPH) Radical Scavenging Method

Antioxidant activity of MUSC 137 extract was examined by measuring radical scavenging ability using DPPH (Sigma–Aldrich). Scavenging activity on DPPH free radicals was determined using previous protocol with minor modifications (Ling et al., 2009). Reduction in the amount of free radicals present was measured via a decrease in absorbance of 515 nm. Volume of 195 μL of 0.016% DPPH ethanolic solution was added to 5 μL of extract solution to make up final volume of 200 μL. For control, DMSO was added in place of extract, while gallic acid was included as positive control. Reactions were carried out in the dark at room temperature for 20 min before measurement with spectrophotometer at 515 nm. DPPH scavenging activity was calculated as follows:

$$DPPH\,\;\,scavenging\,\;activity=\frac{Absorbance\,\;of\,\;control-Absorbance\,\;of\,\;sample\,}{Absorbance\,\;of\,\;control}\times 100\%$$

### Determination of Cytotoxicity Activity of MUSC 137 Using 3-(4,5-Dimethylthazol-2yl)-2,5-Diphenyl Tetrazolium-Bromide (MTT) Assay

For evaluation of cytotoxicity, a variety of human derived cancer cell lines were selected for testing: (a) colon cancer cell, HCT-116, HT-29, Caco-2 and SW480; (b) breast cancer cell, MCF-7; (c) cervical cancer cell, Ca Ski; (d) lung carcinoma cell line, A549; (e) prostate cancer cell, DU 145. Cells were grown in RPMi 1640 supplemented with 10% FBS in humidified incubator (5% CO_2_ in air at 37°C). Cells were seeded into a sterile flat bottom 96-well plate at a density of 5 × 10^3^ cells/well and allowed to adhere overnight. MUSC 137 extract was added to the cells (0–400 μg/mL) and further incubated for 72 h before performing MTT assay. Curcumin (1.56–25 μg/mL) was included as positive control. Twenty microliter of 5 mg/mL of MTT (Sigma) was then added to each well and the plates were incubated at 37°C in a humid atmosphere with 5% CO_2_, 95% air for 4 h. The medium was then gently aspirated, and 100 μL of DMSO was added to dissolve the formazan crystals. The amount of formazan product was determined spectrophotometrically at 570 nm (with 650 nm as reference wavelength) using a microplate reader. The percentage of cell viability was calculated with follows:

$$Percentage\,\;of\,\;cell\,\;viability=\frac{Absorbance\,\;of\,\;treated\,\;cells\,}{Absorbance\,\;of\,\;untreated\,\;cells}\times 100\%$$

The 50% inhibitory concentration of extract (IC_50_) was derived from the plotted graphs for each cell line. VERO cells were used as a normal cell model for determination of selective index (SI). SI value was calculated by dividing IC_50_ of cancer cell line with IC_50_ value of normal cell line.

### Gas Chromatography–Mass Spectrometry (GC–MS) Analysis

Gas chromatography–mass spectrometry analysis was performed as previously described (Ser et al., 2015b). The equipment used was Agilent Technologies 6980N (GC) equipped with 5979 Mass Selective Detector (MS), HP-5MS (5% phenyl methyl siloxane) capillary column of dimensions 30.0 m × 250 μm × 0.25 μm and used helium as carrier gas at 1 mL/min. The column temperature was programmed initially at 40°C for 10 min, followed by an increase of 3°C/min to 250°C and was kept isothermally for 5 min. The MS was operating at 70 eV. The constituents were identified by comparison of their mass spectral data with those from NIST 05 Spectral Library.

### Statistical Analysis

Experiments to investigate antioxidant and cytotoxic activities were done in quadruplicate. Data analysis was performed with SPSS statistical analysis software and the results were expressed as mean ± standard deviation (SD). One-way analysis of variance (ANOVA) was used for comparison of more than two means. A difference was considered statistically significant when *p* ≤ 0.05.

## Results

### Phenotypic Analyses of Strain *S. pluripotens* MUSC 137

Strain MUSC 137 was Gram-positive and aerobic. The strain grew well on ISP2 medium, SA and SCA after 1–2 weeks at 28°C, it grew moderately on Luria Bertani agar, ISP7 medium and nutrient agar, whereas it grew poorly on AIA. The morphological observation of the 15-day-old culture grown on ISP2 medium showed a smooth spore surface, with aerial and vegetative hyphae which was well developed and not fragmented. These morphological characteristics were consistent with its assignment to the genus *Streptomyces* (Williams et al., 1989), (**Figure 2**). The colors of the aerial and substrate mycelium were yellowish gray and brilliant yellow. Growth occurred at pH 5.0–9.0 (optimum pH 5.0–8.0), with 0–6% NaCl tolerance (optimum 0–4%) and at 24–40°C (optimum 28–32°C). MUSC 137 was positive for catalase but negative for hemolytic activity and melanoid pigment production. Hydrolysis of soluble starch and carboxymethylcellulose were positive; but negative for hydrolysis of tributyrin (lipase), casein, chitin, and xylan. Strain MUSC 137 was found to be able to utilize various compounds as carbon sources (**Supplementary Table S1**). The strain was sensitive to ampicillin, ampicillin sulbactam, cefotaxime, cefuroxime, cephalosporin, chloramphenicol, ciprofloxacin, erythromycin, gentamicin, nalidixic acid, Penicillin G, streptomycin, tetracycline, and vancomycin.

### Phylogenetic and Genomic Analyses

The almost-complete 16S rRNA gene sequences were determined for strain MUSC 137 (1491 bp) and aligned manually with the corresponding partial 16S rRNA gene sequences of the type strains of representative members of the genus *Streptomyces* retrieved from GenBank/EMBL/DDBJ databases. A phylogenetic tree constructed based on the 16S prRNA gene sequences showed that strain MUSC 137 (**Figure 1**) formed a distinct clade with type strains *S. pluripotens* MUSC 135^T^, *S. mexicanus* NBRC 100915^T^, *S. cinereospinus* NBRC 15397^T^, *S. coeruleofuscus* NBRC 12757^T^, and *S. chromofuscus* NBRC 12851^T^. Strain MUSC 137 and *S. pluripotens* MUSC 135^T^ formed a distinct subclade at 100% bootstrap value, indicating the high confidence level of the association (**Figure 1**). Strain MUSC 137 exhibited highest 16S rRNA gene sequence similarity to *S. pluripotens* MUSC 135^T^ (100%), followed by *S. cinereospinus* NBRC 15397^T^ (99.18%), which corresponds to 12 nucleotide differences at 1462 locations with gaps, and lower similarity values to *S. mexicanus* NBRC 100915^T^ (99.17%) and *S. coeruleofuscus* NBRC 12757^T^ (98.97%).

The DNA–DNA relatedness values between strain MUSC 137 and *S. pluripotens* MUSC 135^T^ were 83 ± 3.2%, which were significantly higher that 70%, the threshold value for the delineation of bacterial species (Wayne et al., 1987). These results supported the notion that strain MUSC 137 belonged to the same species as *S. pluripotens* MUSC 135^T^. The BOX-PCR results exhibited that strain MUSC 137 presented a unique BOX-PCR fingerprint as compared to MUSC 135^T^ and other closely related type strains (**Supplementary Figure S1**).

### Antioxidant and Cytotoxic Activity of MUSC 137 Extract

**FIGURE 3:**
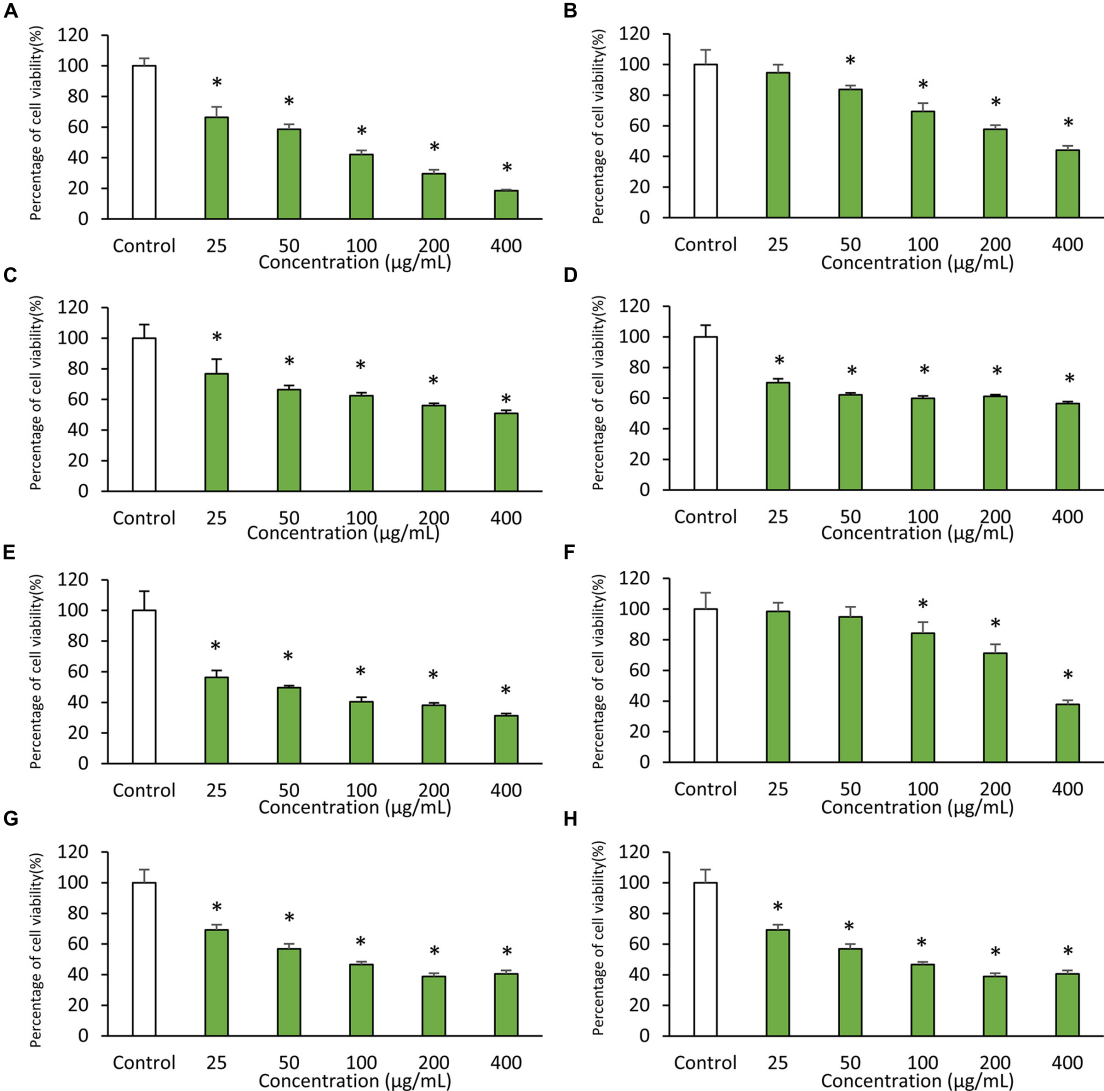
**Cytotoxic activity of MUSC 137 extract against human cancer cell lines.** Cell viability was measured using MTT assay. The graphs show cytotoxicity effects of MUSC 137 extract against **(A)** HCT-116, **(B)** HT-29, **(C)** Caco-2, **(D)** SW480, **(E)** MCF-7, **(F)** Ca Ski, **(G)** A549, **(H)** DU 145. * indicates significant difference when compared with control (*p* value < 0.05).

The results obtained from DPPH free radical scavenging assay showed significant antioxidant activity of MUSC 137 extract with 35.03 ± 3.74% scavenging activity at 2 mg/mL (**Table 1**). In addition, the extract of MUSC 137 displayed variable levels of cytotoxic activity against all the tested human cancer cell lines, with our work recording viability of tested cells ranging from 18.5 to 76.2% (**Figure 3**). A dose response pattern was evaluated using different doses which indicated that the extract may be effective against some cancer cell lines even at the lowest tested concentration (25 μg/mL). The lowest IC_50_ value of MUSC 137 extract was recorded at 61.33 ± 17.10 μg/mL for MCF-7 cells, followed by HCT-116 (83.72 ± 7.17 μg/mL), A549 (147.20 ± 19.23 μg/mL) as shown in **Table 2.** Higher IC_50_ values of MUSC 137 extract were observed for Ca Ski and HT-29, at 300.50 ± 19.76 μg/mL and 300.98 ± 27.52 μg/mL, respectively.

**Table 1:** DPPH free radical scavenging assay showing antioxidant activity of MUSC 137 extract.

Sample	Concentration (μg/mL)	Scavenging activity (%)
MUSC 137 extract	250	1.01 ± 1.30
	500	13.41 ± 3.60
	1000	23.24 ± 5.00
	2000	35.03 ± 3.74
Gallic acid	6.25	47.11 ± 4.15


**Table 2:** IC_50_ value of MUSC 137 extract against several human cancer cell lines.

Cell lines	IC_50_ value (μg/mL)	
	
	MUSC 137 extract	Curcumin
MCF-7	61.33 ± 17.10	6.09 ± 0.59
HCT-116	83.72 ± 7.17	2.63 ± 0.26
A549	147.20 ± 19.23	6.65 ± 0.25
Ca Ski	300.50 ± 19.76	1.85 ± 0.83
HT-29	300.98 ± 27.52	4.60 ± 0.28
DU 145	>400	8.76 ± 1.48
SW480	>400	6.81 ± 0.19
Caco-2	>400	7.23 ± 0.36


*IC_50_ value represents concentration that reduces cell viability to 50%.*

Of particular note, the extract showed higher level of cytotoxicity against some cancer cell lines with low cytotoxic effects in normal cell lines. The calculation of selectivity index (SI) allows comparison of cytotoxicity by dividing IC_50_ value of each cancer cell lines and IC_50_ value of normal cell line. The extract was less cytotoxic to normal cells as indicated by the SI value of 4.22 when compared to MCF-7 cells, followed by HCT-116 with value of 3.09 (**Table 3**).

**Table 3:** Selectivity index (SI) of MUSC 137 extract.

Cell lines	Selectivity index
MCF-7	4.22
HCT-116	3.09
A549	1.76
Ca Ski	0.86
HT-29	0.86
DU 145	N.A.
SW480	N.A.
Caco-2	N.A.


*N.A., not available. SI values were determined by dividing IC_50_ value of VERO cells with IC_50_ value of each cancer cell lines.*

### GC–MS Analysis of MUSC 137 Extract

**FIGURE 4:**
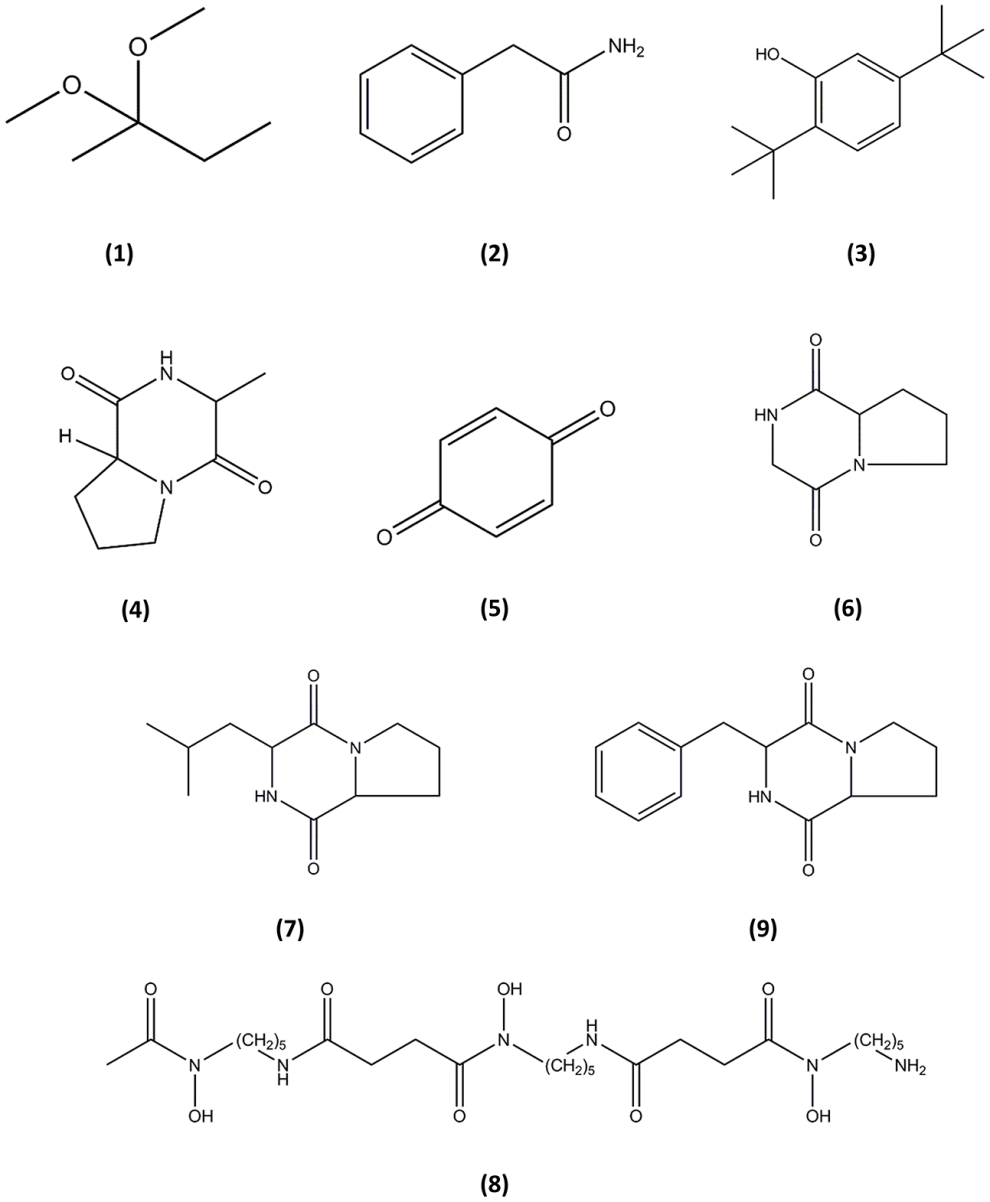
**Chemical structures of the identified compounds from MUSC 137.** (1), 2,2-dimethoxybutane; (2), Benzeneacetamide; (3), Phenol, 2,5-bis(1,1-dimethylethyl)-; (4), (3R,8aS)-3-methyl-1,2,3,4,6,7,8,8a-octahydropyrrolo[1,2-a]pyrazine-1,4-dione; (5), 2,5-cyclohexadiene-1, 4-dione; (6), Pyrrolo[1,2-a]pyrazine-1,4-dione, hexahydro-; (7), 1,4-diaza-2,5-dioxo-3-isobutyl bicyclo[4.3.0]nonane; (8), Deferoxamine; (9), Pyrrolo[1,2-a]pyrazine-1,4-dione, hexahydro-3-(2-phenylmethyl)-.

Gas chromatography–mass spectrometry analysis identified nine compounds present in the methanolic extract of MUSC 137 (**Table 4**) and the chemical structures (**Figure 4**) as 2,2-dimethoxybutane **(1)**, Benzeneacetamide **(2)**, Phenol, 2,5-bis(1,1-dimethylethyl)- **(3)**, (3R,8aS)-3-methyl-1,2,3,4,6,7,8,8a-octahydropyrrolo[1,2-a]pyrazine-1,4-dione **(4)**, 2,5-cyclohexadiene-1,4-dione **(5)**, Pyrrolo[1,2-a]pyrazine-1,4-dione, hexahydro- **(6)**, 1,4-diaza-2,5-dioxo-3-isobutyl bicyclo[4.3.0]nonane **(7)**, Deferoxamine **(8)**, Pyrrolo[1,2-a]pyrazine-1,4-dione, hexahydro-3-(2-phenylmethyl)- **(9)**.

**Table 4:** Compounds identified from MUSC 137 extract through GC–MS.

No	Retention time (min)	Compound	Formula	Molecular weight (MW)	Quality (%)
1	4.781	2,2-dimethoxybutane	C_6_H_14_O_2_	118	83
2	39.358	Benzeneacetamide	C_8_H_9_NO	135	90
3	44.457	Phenol, 2,5-bis(1,1-dimethylethyl)-	C_14_H_22_O	206	90
4	51.540	(3R,8aS)-3-methyl-1,2,3,4,6,7,8,8a-octahydropyrrolo[1,2-a]pyrazine-1,4-dione	C_8_H_12_N_2_O_2_	168	86
5	51.878	2,5-cyclohexadiene-1,4-dione	C_6_H_4_O_4_	140	56
6	53.028	Pyrrolo[1,2-a]pyrazine-1,4-dione, hexahydro-	C_7_H_10_N_2_O_2_	154	97
7	58.476	1,4-diaza-2,5-dioxo-3-isobutyl bicyclo[4.3.0]nonane	C_11_H_18_N_2_O_2_	210	72
8	71.556	Deferoxamine	C_25_H_48_N_6_O_8_	560	81
9	72.054	Pyrrolo[1,2-a]pyrazine-1,4-dione, hexahydro-3-(2-phenylmethyl)-	C_14_H1_6_N_2_O_2_	244	58


## Discussion

Microorganisms have served as a promising source for the majority of the drugs in use today (Berdy, 2005; Azman et al., 2015). Their ability to produce a diverse array of bioactive small molecule natural products has made them among the key players in the search for novel therapeutic or preventive agents against diseases including cancer, neurodegenerative diseases and diabetes (Berdy, 2005; Chin et al., 2006). The phylum *Actinobacteria* produces approximately 10,000 clinically important compounds, of which about 75% are isolated from the genus *Streptomyces* (Watve et al., 2001; Berdy, 2005). These filamentous bacteria under the genus *Streptomyces* are economically important from a biotechnological and pharmacological point of view as they seem to be a virtually inexhaustible source of bioactive secondary metabolites with potent antibacterial, antifungal, antitumor and antioxidant activity (Kim et al., 2008; Olano et al., 2009a; Saurav and Kannabiran, 2012; Wang et al., 2013). In the current study, *S. pluripotens* MUSC 137 was isolated from a soil sample from a mangrove forest in Pahang, Peninsular Malaysia. Results from phylogenetic and genomic analyses indicated that strain MUSC 137 is closely related to *S. pluripotens* MUSC 135^T^ as they formed a distinct subclade at 100% bootstrap value. Furthermore, the DNA–DNA relatedness values between MUSC 137 and MUSC 135^T^ were 83 ± 3.2%, which were significantly higher that 70%, the threshold value for the delineation of bacterial species (Wayne et al., 1987). Also the BOX-PCR results exhibited that strain MUSC 137 presented a unique BOX-PCR fingerprint as compared to MUSC 135^T^ and other closely related type strains, indicating that MUSC 137 and MUSC 135^T^ are not clones. Overall, these results support the notion that strain MUSC 137 belonged to the same species as *S. pluripotens* MUSC 135^T^ but has sufficient unique characteristics to be designated as a separate strain.

Cancer initiation and progression is associated with accumulation of free radicals or oxidative stress (Reuter et al., 2010). In general, free radicals cause various modifications or cause damage to biological macromolecules such as proteins, lipids and DNA. These deleterious effects then compromise functioning of important pathways including the DNA repair system which eventually results in an increased mutation rate (Pacifici and Davies, 1991; Bartsch and Nair, 2006). As antioxidants are capable of scavenging free radicals, these molecules could potentially affect the activation of signaling pathways which are crucial for survival of cancer cells (Lopaczynski and Zeisel, 2001; Chen et al., 2009). The bioactive potential of *S. pluripotens* strain MUSC 137 was explored using DPPH free radical scavenging assay to determine antioxidant activity. In the presence of antioxidants, the odd electron of DPPH radical is paired off which causes discoloration of the solution. This important characteristic has enabled researchers to assess the antioxidant strength of the sample(s) of interest. In this current study, DPPH assay revealed significant antioxidant activity of MUSC 137 extract (**Table 1**), which suggests that the strain may produce valuable compound(s) that could potentially reduce cancer occurrence and be further developed as chemopreventive drugs.

The cytotoxicity of MUSC 137 extract against several human cancer cell lines was examined with MTT assay. This tetrazolium-based colorimetric assay measures only *in vitro* living cells and is often employed in cytotoxicity studies to evaluate drug efficiency (Rubinstein et al., 1990; Ciapetti et al., 1993; Fotakis and Timbrell, 2006; Chan et al., 2015). The extract of MUSC 137 displayed varying levels of cytotoxicity against the tested cancer cell lines. MCF-7 was found to be the most vulnerable to the treatment extract with the lowest IC_50_ (61.33 ± 17.10 μg/mL), followed by HCT-116 (83.72 ± 7.17 μg/mL) and A549 (147.20 ± 19.23 μg/mL). Among the tested colon cancer cell lines, the treatment of MUSC 137 extract at the highest tested concentration (400 μg/mL) showed strongest growth inhibition against HCT-116 (18.5 ± 7.8%), while higher percentages of cell viability were still observed in HT-29 (44.1 ± 2.9%), Caco-2 (50.8 ± 2.1%), and SW480 (56.4 ± 1.3%). The tested colon cancer cell lines are known to possess dysfunctional p53 tumor protein, except HCT-116. Therefore, the varied levels of cytotoxic activity among these colon cancer cell lines exhibited by extract of MUSC 137 might be due to the p53 tumor suppressor protein status of these cancer cell lines (Petitjean et al., 2007; Goh et al., 2014). These findings have led to the speculation that MUSC 137 extract might be able to induce cancer cell death via p53 dependent cell death signaling pathway. Elucidation of the underlying mechanism of MUSC 137 extract on cancer cell lines may be a valuable area of future research as these information could potentially assist in its development as chemopreventive drug.

An ideal chemotherapy drug should have high specificity and should be able to discriminate between cancer and normal cells. However, many of the anticancer drugs in use are still lacking in the drug specificity as they kill both cancer cells as well as normal cells (Chabner and Roberts, 2005; Chari, 2007; Fernandez, 2014). Tremendous efforts have been invested to search for novel chemotherapy drugs with high potency and specificity. This current study investigating specificity of MUSC 137 extract revealed that the extract was less toxic against normal cell lines when compared to MCF-7 and HCT-116 cancer cell lines as shown by the SI value of 4.22 and 3.09, respectively. These crucial findings have provided new insight into the cytotoxic potential of MUSC 137 against cancer cells, particularly against MCF-7 and HCT-116 with high specificity. Further analysis investigating the target action of the bioactive compounds could provide helpful information for future development as an anticancer drug. In summary, the results of both the DPPH assay and *in vitro* anticancer screening suggests the presence of potent biologically active compounds in the extract of MUSC 137, which then prompted subsequent chemical analysis using GC–MS to identify the chemical constituents present in the extract.

A powerful analytical tool, GC–MS combines the separation power of GC and detection power of MS, generating robust and reliable data (Hites, 1997; Stein, 1999). GC–MS plays an important role in natural product discovery, including bioactive compounds derived from *Streptomyces* species (Pollak and Berger, 1996; Sudha and Masilamani, 2012; Ara et al., 2014; Jog et al., 2014). In the current study, GC–MS detected nine compounds in the extract of MUSC 137, with the majority of the compounds having been detected in marine-derived microorganisms including *Streptomyces* species (Hong et al., 2008; Gao et al., 2014; Narendhran et al., 2014). The analysis detected several members of pyrrolizidines which are known to exhibit a diverse array of bioactivities including antimicrobial, antitumor, anti-angiogenesis and antioxidant activities (Olano et al., 2009b; Melo et al., 2014; Robertson and Stevens, 2014). The pyrrolizidines in the extract of MUSC 137 include 1,4-diaza-2,5-dioxo-3-isobutyl bicyclo[4.3.0]nonane, Pyrrolo[1,2-a]pyrazine-1,4-dione, hexahydro-3-(2-phenylmethyl)-, Pyrrolo[1,2-a]pyrazine-1,4-dione, hexahydro- and (3R,8aS)-3-methyl-1,2,3,4,6,7,8,8a-octahydropyrrolo[1,2-a]pyrazine-1,4-dione. As a matter of fact, Pyrrolo[1,2-a]pyrazine-1,4-dione, hexahydro-3-(2-phenylmethyl)- has been shown to inhibit expression of serine/threonine kinase Akt which involves in regulation cell proliferation and apoptosis (Hong et al., 2008). Furthermore, the chemical compound 1,4-diaza-2,5-dioxo-3-isobutyl bicyclo[4.3.0]nonane has also been associated with the cytotoxic activity observed on human cervical cancer cell line (Narendhran et al., 2014). Therefore, the presence of these compounds in the extract of MUSC 137 could account for the observed cytotoxicity against the tested cancer cell lines.

In addition, GC–MS analysis also detected compounds that may account for the antioxidant activity observed. In the current study, Pyrrolo[1,2-a]pyrazine-1,4-dione, hexahydro- and (3R,8aS)-3-methyl-1,2,3,4,6,7,8,8a-octahydropyrrolo[1,2-a]pyrazine-1,4-dione were also found in other *Streptomyces* and *Bacillus* strains which exhibited antioxidant activity (Gopi et al., 2014; Ser et al., 2015b). As antioxidants are suggested to affect survival of cancer cells (Lopaczynski and Zeisel, 2001; Chen et al., 2009), these pyrrolizidines with antioxidant activity in MUSC 137 could be contributing to the observed cytotoxic effects. Besides pyrrolizidines, GC–MS has also revealed the presence of deferoxamine in MUSC 137. Deferoxamine (with the prescription name of Desferal) is listed on World Health Organization’s List of Essential Medicines due to its medical importance as iron chelator and to protect against iron-induced oxidative stress (World Health Organization [WHO], 2015). This trihydroxamate molecule can reduce accumulation of free radicals and inhibit growth of various cancer cell lines and tumors (Tomoyasu et al., 1992; Shimoni et al., 1994; Melillo et al., 1997; Buss et al., 2004; Yamasaki et al., 2011; Salis et al., 2014). The detection of deferoxamine in MUSC 137 might be associated with the antioxidant activity. Also, it may affect the expression of crucial genes and signaling pathways responsible for the survival of cancer cells, subsequently leading to a reduction in viability of cancer cell as shown in MTT assay.

## Conclusion

This study has revealed the biopharmaceutical potential possessed by *S. pluripotens* strain MUSC 137 as this strain has demonstrated its ability to produce bioactive compounds which may account for its antioxidant and cytotoxic activities. Future studies should focus on the elucidation of mechanism action of cell death to provide more convincing evidence of selective cytotoxic activity observed and work investigating this phenomenon is in fact currently underway.

## Author Contributions

H-LS, B-HG, and L-HL contributed to the data analyses and writing of the manuscript. N-SA, W-FY, and K-GC contributed by providing vital technical support for the project. K-GC, B-HG, and L-HL contributed to the inception of the experimental design and funding of the project.

## Conflict of Interest Statement

The authors declare that the research was conducted in the absence of any commercial or financial relationships that could be construed as a potential conflict of interest.

## Footnotes

1 http://www.bacterio.cict.fr/.

2 http://eztaxon-e.ezbiocloud.net/
