# Supplementary material for: Evaluation of Antioxidative and Cytotoxic Activities of Streptomyces pluripotens MUSC 137 Isolated from Mangrove Soil in Malaysia
Source: Front Microbiol. 2015 Dec 16;6:1398. doi: 10.3389/fmicb.2015.01398 (PMC4679926; doi:10.3389/fmicb.2015.01398)
Supplement: Supplementary file 1 [file Data_Sheet_1.DOCX]

***Supplementary Material***

**Evaluation of antioxidative and cytotoxic activities of Streptomyces pluripotens MUSC 137 isolated from mangrove soil in Malaysia**

**Hooi-Leng Ser^1^,** **Nurul-Syakima Ab Mutalib^2^, Wai-Fong Yin^3^, Kok-Gan Chan^3^, Bey-Hing Goh^1*^, Learn-Han Lee^1*^**

^1^Biomedical Research Laboratory, Jeffrey Cheah School of Medicine and Health Sciences, Monash University Malaysia, 47500 Bandar Sunway, Selangor Darul Ehsan, Malaysia

^2^UKM Medical Molecular Biology Institute (UMBI), UKM Medical Centre, Kuala Lumpur, Malaysia

^3^Division of Genetics and Molecular Biology, Institute of Biological Sciences, Faculty of Science, University of Malaya, 50603 Kuala Lumpur, Malaysia

*** Correspondence:** Learn-Han Lee, Biomedical Research Laboratory, Jeffrey Cheah School of Medicine and Health Sciences, Monash University Malaysia, 47500 Bandar Sunway, Selangor Darul Ehsan, Malaysia. E-mail: lee.learn.han@monash.edu; [leelearnhan@yahoo.com](mailto:leelearnhan@yahoo.com). Bey-Hing Goh, Jeffrey Cheah School of Medicine and Health Sciences, Monash University Malaysia, 47500 Bandar Sunway, Selangor Darul Ehsan, Malaysia. E-mail: goh.bey.hing@monash.edu.

## Supplementary Figure

**Supplementary Fig. S1.** BOX-PCR comparison of strain MUSC 137 and the closest related type strains.


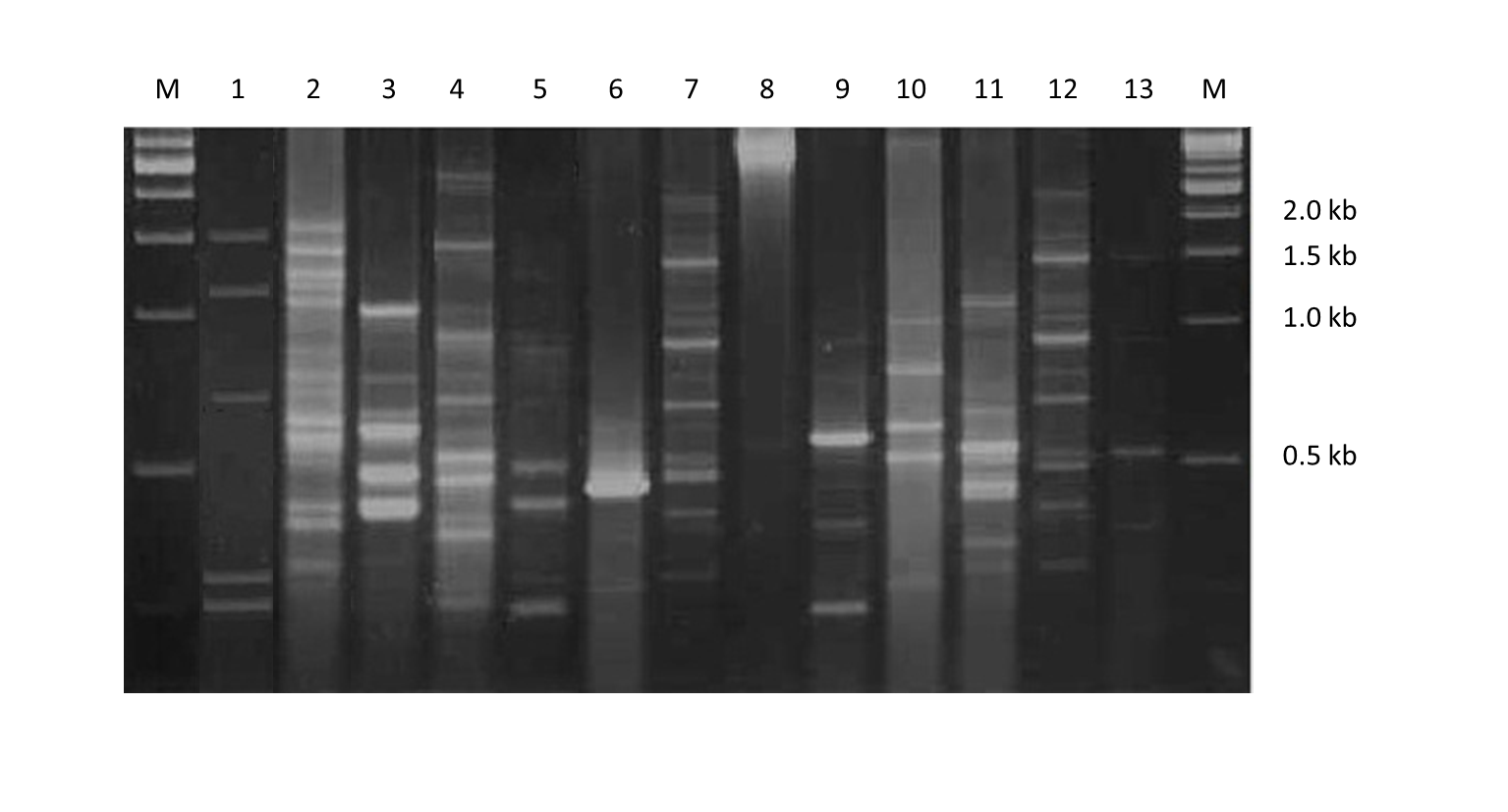
Lanes: 1, *Streptomyces pluripotens* sp. nov. MUSC 137; 2, *Streptomyces pluripotens* sp. nov. MUSC 135^T^; 3, *Streptomyces cinereospinus* NBRC 15397^T^; 4, *Streptomyces coeruleofuscus* NBRC 12757^T^; 5, *Streptomyces mexicanus* NBRC 100915^T^; 6, *Streptomyces chromofuscus* NBRC 12851^T^; 7, *Streptomyces nogalater* NBRC 12851^T^; 8, *Streptomyces flavofungini* NBRC 13371^T^; 9, *Streptomyces indiaensis* NBRC 13964^T^; 10, *Streptomyces koyangensis* NBRC 100598^T^; 11, *Streptomyces thermocarboxydovorans* NBRC 16324^T^; 12, *Streptomyces glomeratus* NBRC 15898^T^; 13, *Streptomyces rameus* NBRC 16196^T^. M, GeneRuler 1kb DNA ladder marker.

## Supplementary Table

**Supplementary Table S1. Carbon utilization and chemical sensitivity of MUSC 137**

| Carbon source | Growth |
| --- | --- |
| Dextrin | - |
| D-maltose | + |
| D-trehalose | + |
| D-cellobiose | + |
| Gentiobiose | + |
| Sucrose | - |
| D-turanose | + |
| Stachyose | + |
| D-raffinose | - |
| α-D-lactose | - |
| D-melibiose | + |
| β-methyl-D-glucoside | + |
| D-salicin | + |
| N-acetyl-D-glucosamine | + |
| N-acetyl-β-D-mannosamine | + |
| N-acetyl-D-galactosamine | + |
| N-acetyl-neuraminic acid | + |
| α-D-glucose | - |
| D-mannose | + |
| D-fructose | - |
| D-galactose | + |
| 3-methyl glucose | + |
| D-fucose | + |
| L-fucose | - |
| L-rhamnose | + |
| Inosine | + |
| D-sorbitol | - |
| D-mannitol | - |
| D-arabitol | - |
| Myo-inositol | - |
| Glycerol | + |
| D-glucose-6-PO_4_ | + |
| D-fructose-6-PO_4_ | + |
| D-aspartic acid | + |
| D-serine | + |
| Gelatin | - |
| Glycyl-L-proline | - |
| L-alanine | - |
| L-arginine | - |
| L-aspartic acid | + |
| L-glutamic acid | - |
| L-histidine | + |
| L-pyroglutamic acid | - |
| L-serine | + |
| Pectin | - |
| D-galacturonic acid | - |
| L-galactonic acid lactone | + |
| D-gluconic acid | - |
| D-glucuronic acid | - |
| Glucuronamide | + |
| Mucic acid | - |
| Quinic acid | - |
| D-saccharic acid | + |
| p-hydroxy-phenylacetic acid | - |
| methyl pyruvate | - |
| D-lactic acid methyl ester | + |
| L-lactic acid | + |
| Citric acid | + |
| α-keto-glutaric acid | + |
| D-malic acid | - |
| L-malic acid | - |
| Bromo-succinic acid | + |
| Tween 40 | + |
| γ-amino-butyric acid | + |
| α-hydroxy-butyric acid | + |
| β-hydroxy-D,L-butyric acid | + |
| α-keto-butyric acid | - |
| Acetoacetic acid | + |
| Propionic acid | - |
| Acetic acid | + |
| Formic acid | + |
| **Chemical sensitivity** |  |
| 1% sodium lactate | + |
| Fusidic acid | + |
| D-serine | + |
| Troleandomycin | + |
| Rifamycin RV | + |
| Minocycline | + |
| Lincomycin | + |
| Guanidine HCl | + |
| Niaproof 4 | + |
| Vancomycin | + |
| Tetrazolium violet | + |
| Tetrazolium blue | + |
| Nalixidic acid | + |
| Lithium chloride | + |
| Potassium tellurite | - |
| Aztreonam | + |
| Sodium butyrate | + |
| Sodium bromate | + |
